# Supplementary material for: Leveraging machine learning to uncover multi-pathogen infection dynamics across co-distributed frog families
Source: PeerJ. 2025 Jan 29;13:e18901. doi: 10.7717/peerj.18901 (PMC11786709; doi:10.7717/peerj.18901)
Supplement: Supplemental Information 2 [file peerj-13-18901-s002.docx]

Supplemental tables below last updated 8/1/2024 for CFPCS manuscript submission by DLFW.

**List of Supplementary Tables**

**Table S1. Sample information for all individuals (separate excel spreadsheet).**

**Table S2. Bd prevalence across sampling with 95% Confidence Intervals**

**Table S3. Pr prevalence across sampling with 95% Confidence Intervals**

**Table S4. Rv prevalence across sampling with 95% Confidence Intervals**

**Table S5. PCA loadings and interpretations relating to temperature.**

**Table S6. PCA loadings and interpretations relating to precipitation.**

**Table S7. Pathogen infection prevalence by age class**

**Table S8. Pathogen infection prevalence by sex class**

**Table S9. Pathogen infection prevalence by tissue type tested**

**Table S10. Parametric and non-parametric results when only considering samples derived from external tissues**

**Table S11. Pathogen infection intensity by family and analysis of variance results**

**Table S12. Pathogen infection intensity by species.**

**Table S13. Pathogen infection intensity by age.**

**Table S14. Pathogen infection intensity by sex.**

| **Family** | **N Family** | **Genus** | **Species binomial** | **N individuals** | **Bd+** | **Bd-** | **% Pos** | **Fam Pos** | **% Fam Pos** | **95% CI (Logit)** | |
| --- | --- | --- | --- | --- | --- | --- | --- | --- | --- | --- | --- |
| Bufonidae | 320 | *Anaxyrus* | *americanus* | 108 | 19 | 89 | 17.6 | 23 | 7.19 | **4.82-10.6%** | **11.5-25.9%** |
|  |  |  | *fowleri* | 96 | 2 | 94 | 2.1 |  |  |  | **0.521-7.94%** |
|  |  |  | *terrestris* | 90 | 2 | 88 | 2.2 |  |  |  | **0.556-8.45%** |
|  |  |  | *woodhousii* | 26 | 0 | 26 | 0.0 |  |  |  | **0-13.2%** |
| Hylidae | 456 | *Hyla* | *chryss-vers complex* | 140 | 6 | 134 | 4.3 | 32 | 7.02 | **5-9.76%** | **1.94-9.21%** |
|  |  |  | *cinerea* | 125 | 5 | 120 | 4.0 |  |  |  | **1.67-9.25%** |
|  |  |  | *squirella* | 97 | 1 | 96 | 1.0 |  |  |  | **0.145-6.95%** |
|  |  | *Pseudacris* | *crucifer* | 94 | 20 | 74 | 21.3 |  |  |  | **14.2-30.7%** |
| Ranidae | 505 | *Rana* | *catesbeiana* | 104 | 38 | 66 | 36.5 | 161 | 31.9 | **26.6-34.4%** | **27.8-46.2%** |
|  |  |  | *clamitans* | 109 | 48 | 61 | 44.0 |  |  |  | **35-53.5%** |
|  |  |  | *pipiens* | 80 | 17 | 63 | 21.3 |  |  |  | **13.6-31.6%** |
|  |  |  | *sphenocephala* | 212 | 58 | 154 | 27.4 |  |  |  | **21.8-33.7%** |
| **Total** | **1281** |  |  | **Total** | **216** | **1065** | **16.8** | **216** | **16.8** | **14.9-19%** | |

**Supplemental Table S2. Taxonomic distribution of Bd screening effort and prevalence.** The number of individuals screened and found to be infected and uninfected for Bd with associated proportion positive and confidence intervals across taxonomy. The associated 95% confidence intervals were calculated using the logit method.

| **Family** | **N Family** | **Genus** | **Species binomial** | **N individuals** | **Pr+** | **Pr-** | **% Pos** | **Fam Pos** | **% Fam Pos** | **95% CI (logit)** | |
| --- | --- | --- | --- | --- | --- | --- | --- | --- | --- | --- | --- |
| Bufonidae | 308 | *Anaxyrus* | *americanus* | 105 | 2 | 103 | 1.90 | 2 | 0.65 | **0.162-2.56%** | **0.477-7.29%** |
|  |  |  | *fowleri* | 87 | 0 | 87 | 0.00 |  |  |  | **0-4.15%** |
|  |  |  | *terrestris* | 90 | 0 | 90 | 0.00 |  |  |  | **0-4.02%** |
|  |  |  | *woodhousii* | 26 | 0 | 26 | 0.00 |  |  |  | **0-13.2%** |
| Hylidae | 443 | *Hyla* | *chry-vers complex* | 139 | 2 | 137 | 1.44 | 4 | 0.90 | **0.339-2.38%** | **0.36-5.57%** |
|  |  |  | *cinerea* | 122 | 1 | 121 | 0.82 |  |  |  | **0.115-5.58%** |
|  |  |  | *squirella* | 95 | 1 | 94 | 1.05 |  |  |  | **0.148-7.1%** |
|  |  | *Pseudacris* | *crucifer* | 87 | 0 | 87 | 0.00 |  |  |  | **0-4.15%** |
| Ranidae | 473 | *Rana* | *catesbeiana* | 97 | 1 | 96 | 1.03 | 7 | 1.48 | **0.71-3.07%** | **0.145-6.95%** |
|  |  |  | *clamitans* | 95 | 1 | 94 | 1.05 |  |  |  | **0.148-7.09%** |
|  |  |  | *pipiens* | 70 | 0 | 70 | 0.00 |  |  |  | **0-5.13%** |
|  |  |  | *sphenocephala* | 211 | 5 | 206 | 2.37 |  |  |  | **1-5.57%** |
| **Total** | **1224** |  |  | **Total** | **13** | **1211** | **1.06** | **13** | **1.06** | **0.618-1.82%** | |

**Supplemental Table S3. Taxonomic distribution of Pr screening effort and prevalence.** The number of individuals screened and found to be infected and uninfected for Pr with associated proportion positive and confidence intervals across taxonomy. The associated 95% confidence intervals were calculated using the logit method.

| **Family** | **N Family** | **Genus** | **Species binomial** | **N individuals** | **Rv+** | **Rv-** | **% Pos** | **Fam Pos** | **% Fam Pos** | **95% CI (logit)** | |
| --- | --- | --- | --- | --- | --- | --- | --- | --- | --- | --- | --- |
| Bufonidae | 301 | *Anaxyrus* | *americanus* | 105 | 2 | 103 | 1.90 | 3 | 1.00 | **0.321-3.04%** | **0.477-7.29%** |
|  |  |  | *fowleri* | 84 | 1 | 83 | 1.19 |  |  |  | **0.167-7.97%** |
|  |  |  | *terrestris* | 86 | 0 | 86 | 0.00 |  |  |  | **0-4.2%** |
|  |  |  | *woodhousii* | 26 | 0 | 26 | 0.00 |  |  |  | **0-13.2%** |
| Hylidae | 418 | *Hyla* | *chrys-vers complex* | 132 | 1 | 131 | 0.76 | 8 | 1.91 | **0.96-3.85%** | **0.-5.18%** |
|  |  |  | *cinerea* | 115 | 4 | 111 | 3.48 |  |  |  | **1.31-8.9%** |
|  |  |  | *squirella* | 89 | 1 | 88 | 1.12 |  |  |  | **0.158-7.54%** |
|  |  | *Pseudacris* | *crucifer* | 82 | 2 | 80 | 2.44 |  |  |  | **0.611-9.23%** |
| Ranidae | 468 | *Rana* | *catesbeiana* | 96 | 19 | 77 | 19.79 | 41 | 8.76 | **6.51-11.7%** | **13-29%** |
|  |  |  | *clamitans* | 95 | 12 | 83 | 12.63 |  |  |  | **6.08-17.6%** |
|  |  |  | *pipiens* | 70 | 0 | 70 | 0.00 |  |  |  | **0-5.13%** |
|  |  |  | *sphenocephala* | 207 | 10 | 197 | 4.83 |  |  |  | **2.62-8.74%** |
| **Total** | **1187** |  |  | **Total** | **52** | **1135** | 4.38 | **52** | **4.38** | **3.35-5.7%** | |

**Supplemental Table S4. Taxonomic distribution of Rv screening effort and prevalence.** The number of individuals screened and found to be infected and uninfected for Rv with associated proportion positive and confidence intervals across taxonomy. The associated 95% confidence intervals were calculated using the logit method.

| **PCA Loadings** | | |
| --- | --- | --- |
| *Temperature* | | |
| **Dimension 1 (PC1)** | **74.9% variation captured** | |
| *Contributing variables* | *Contribution of rows* | *Interpretation as TPC1 increases* |
| BIO11: Temp of Coldest Quarter | 0.341 | Mean temps of coldest quarter increase |
| BIO6: Min Temp of Coldest Month | 0.34 | Minimum temps of coldest month increase |
| BIO1: Annual Mean Temp | 0.339 | Mean annual temps increase |
| BIO4: Temp seasonality | -0.332 | Less variable temperatures across the year |
| BIO10: Mean Temp of Warmest Quarter | 0.325 | Mean temps of warmest quarter increase |
| BIO7: Temp annual range | -0.319 | Temp annual range decrease |
| BIO9: Mean Temp of Driest Quarter | 0.316 | Mean temps of driest quarter increase |
| BIO3: Isothermality | 0.306 | More stable temperatures (day-night; summer-winter) |
|  |  |  |
| **Dimension 2 (PC2)** | **13.8% variation further captured** | |
| *Contributing variables* | *Contribution of rows* | *Interpretation as TPC2 increases* |
| BIO2: Mean Diurnal Range | 0.733 | More extreme daily temperature fluctuation |
| BIO5: Max Temp of Warmest Month | 0.391 | Max temp of warmest month increase |
| BIO8: Mean Temp of Wettest Quarter | 0.359 | Mean temp of wet quarter decrease |

**Supplemental Table S5 First and second dimension PCA loadings and interpretations relating to temperature (TPC1-2).** Contributing variables to PC axes listed in descending order. Contribution of rows shows the relative contribution and direction of the variable relationship to an increasing PC axis.

| **PCA Loadings** | | |
| --- | --- | --- |
| *Precipitation* | | |
| **Dimension 1 (PC1)** | **69.3% variation explained** | |
| *Contributing variables* | *Contribution of rows* | *Interpretation as PPC1 increases* |
| BIO12: Annual Precipitation | 0.421 | Higher precipitation overall |
| BIO17: Precipitation during driest quarter | 0.387 | Wetter dry quarter |
| BIO19: Precipitation of coldest quarter | 0.385 | Wetter cold quarter |
| BIO14: Precipitation of driest month | 0.374 | Wetter dry months |
| BIO16 Precipitation of wettest quarter | 0.363 | Wetter wet quarter |
|  |  |  |
| **Dimension 2 (PC2)** | **26.5% variation further explained** | |
| *Contributing variables* | *Contribution of rows* | *Interpretation as PPC2 increases* |
| BIO15: Seasonality coefficient | -0.631 | Less variable rainfall |
| BIO18: Precipitation of warmest quarter | -0.382 | Less precipitation during summer |
| BIO13 Precipitation of Wettest Month | -0.364 | Less precipitation during wet season |

**Supplemental Table S6 First and second dimension PCA loadings and interpretations relating to precipitation (PPC1-2).** Contributing variables to PC axes listed in descending order. Contribution of rows shows the relative contribution and direction of the variable relationship to an increasing PC axis.

| **Pathogen** | **N tested** | **Age class** | **+** | **-** | **% Pos** | **95% CI (logit)** | **Pearson's chi-squared statistic and p-value (α = 0.05)** |
| --- | --- | --- | --- | --- | --- | --- | --- |
| Bd | 1,281 | adult | 145 | 717 | 16.8% | **14.5-19.5%** | χ2 (1, N = 1,010) = 2.25, *p* = 0.134 |
|  |  | juvenile | 33 | 115 | 22.3% | **16.3-29.7%** |  |
|  |  | larval | 0 | 8 | 0.0% | **0-36.9%** |  |
|  |  | unknown | 38 | 225 | 14.4% |  |  |
| Pr | 1,224 | adult | 8 | 818 | 1.0% | **0.485-1.92%** | χ2 (1, N = 961) = 4.0e-30, *p* = 1 |
|  |  | juvenile | 1 | 134 | 0.7% | **0.104-5.07%** |  |
|  |  | larval | 0 | 8 | 0.0% | **0-36.9%** |  |
|  |  | unknown | 4 | 251 | 1.6% |  |  |
| Rv | 1,187 | adult | 24 | 776 | 3.0% | **2.02-4.43%** | χ2 (1, N = 935) = 14.3, *p* < 0.001 |
|  |  | juvenile | 14 | 121 | 10.4% | **6.24-16.8%** |  |
|  |  | larval | 0 | 8 | 0.0% | **0-36.9%** |  |
|  |  | unknown | 14 | 230 | 5.7% |  |  |

**Supplemental Table S7. Summary of pathogen screening effort, prevalence, and statistical significance across age class.** The number of individuals screened and found to be infected and uninfected for each pathogen with associated proportion positive and confidence intervals across age class. The associated 95% confidence intervals were calculated using the logit method. Pearson’s chi-squared results comparing pathogen prevalence between adult and juvenile frogs with a significance at *p* < 0.05.

| **Pathogen** | **N tested** | **Sex** | **+** | **-** | **% Pos** | **95% CI (logit)** | **Pearson's chi-squared statistic and p-value (α = 0.05)** |
| --- | --- | --- | --- | --- | --- | --- | --- |
| Bd | 1,281 | female | 49 | 256 | 39.1% | **12.4-20.6%** | χ2 (1, N = 744) = 0.011, *p* = 0.918 |
|  |  | male | 73 | 366 | 27.3% | **13.4-20.4%** |  |
|  |  | unknown | 94 | 443 | 22.6% |  |  |
| Pr | 1,224 | female | 2 | 292 | 34.2% | **0.17-2.68%** | χ2 (1, N = 720) = 0.308, *p* = 0.58 |
|  |  | male | 6 | 420 | 23.8% | **0.634-3.19%** |  |
|  |  | unknown | 5 | 499 | 20.0% |  |  |
| Rv | 1,187 | female | 6 | 287 | 34.8% | **0.923-4.48%** | χ2 (1, N = 695) = 0.278, *p* = 0.599 |
|  |  | male | 12 | 390 | 25.6% | **1.7-5.18%** |  |
|  |  | unknown | 34 | 458 | 21.8% |  |  |

**Supplemental Table S8. Summary of pathogen screening effort, prevalence, and statistical significance across sex.** The number of individuals screened and found to be infected and uninfected for each pathogen with associated proportion positive and confidence intervals across sexes. The associated 95% confidence intervals were calculated using the logit method. Pearson’s chi-squared results comparing pathogen prevalence between female and male frogs with a significance at *p* < 0.05.

| **Pathogen** | **N tested** | **Tissue** | **+** | **-** | **% Pos** | **95% CI (logit)** | **Pearson's chi-squared statistic and p-value (α = 0.05) with “combination” + “external” combined** |
| --- | --- | --- | --- | --- | --- | --- | --- |
| Bd | 1,281 | combination | 136 | 461 | 22.78% | **19.5-26.3%** | χ2 (1, N = 1,208) = 59.7, *p* < 0.001 |
|  |  | external | 62 | 186 | 25.00% | **20.0-30.8%** |  |
|  |  | internal | 17 | 346 | 4.68% | **2.93-7.40%** |  |
|  |  | unknown | 1 | 72 | 1.37% |  |  |
| Pr | 1,224 | combination | 7 | 559 | 1.24% | **0.059-2.57%** | χ2 (1, N = 1,151) = 0.125, *p* = 0.724 |
|  |  | external | 1 | 237 | 0.42% | **0.059-2.92%** |  |
|  |  | internal | 5 | 342 | 1.44% | **0.601-3.41%** |  |
|  |  | unknown | 0 | 73 | 0.00% |  |  |
| Rv | 1,187 | combination | 29 | 533 | 5.16% | **3.61-7.33%** | χ2 (1, N = 1,115) = 0.025, *p* = 0.874 |
|  |  | external | 8 | 217 | 3.56% | **1.79-6.95%** |  |
|  |  | internal | 14 | 314 | 4.27% | **2.30-6.41%** |  |
|  |  | unknown | 1 | 71 | 1.39% |  |  |

**Supplemental Table S9. Summary of Bd screening effort, prevalence, and statistical significance across tissue types.** The number of individuals screened and found to be infected and uninfected for each pathogen with associated proportion positive and confidence intervals across tissue types. The associated 95% confidence intervals were calculated using the logit method. Pearson’s chi-squared results comparing pathogen prevalence between combination, external, and internal samples with a significance at *p* < 0.05.

| *Bd Status* | | | |
| --- | --- | --- | --- |
|  | *p* value | df | χ2 |
| **Family** | **< 0.001** | 2 | 123 |
| **Species** | **< 0.001** | 10 | 153 |
| **Age class** | 0.371 | 1 | 0.8 |
| **Sex** | 1 | 1 | <0.001 |
|  | *p* value | df | t stat |
| **Latitude** | 0.221 | 843 | -1.23 |
| **Longitude** | **<0.001** | 843 | -3.76 |
| **Log₁₀(Elevation)** | 0.064 | 388 | 1.85 |
| **TPC1** | 0.088 | 843 | 1.71 |
| **TPC2** | **< 0.001** | 294 | 4.41 |
| **PPC1** | 0.102 | 372 | -1.64 |
| **PPC2** | 0.169 | 843 | -1.38 |
| *Bd Intensity* | | | |
|  | *p* value | df | F/t stat |
| **Family** | **0.007** | 2,177 | (F) 5.15 |
| **Species** | **>0.001** | 9,16.5 | (F) 20 |
| **Age class** | **>0.001** | 147 | (t) -3.51 |
| **Sex** | 0.562 | 98 | (t) -0.582 |

**Supplemental Table S10 Summary of parametric and non-parametric statistical analyses excluding internal only samples.** Values from tests of Bd status across 11 factors are shown at the top and tests of Bd intensity across 6 factors are shown on the bottom. The test statistic (χ2, t stat, or F stat), degrees of freedom (df), and *p* value are shown for Bd. Bold text denotes significance (*p* < 0.05).

| **Pathogen group** | **Min** | **1^st^ Quarter** | **Median** | **Mean** | **3^rd^ Quarter** | **Max** | **p-values (α = 0.05)** | **post-hoc p-value (Bd p-value: α = 0.05; Rv Bonferroni p-value: α = 0.017)** |
| --- | --- | --- | --- | --- | --- | --- | --- | --- |
| ***Bd total n = 192*** | 0.031 | 0.851 | 1.638 | 1.808 | 2.658 | 4.618 | Bd One-Way Welch ANOVA: F(2, 189) = 4.69, p-value = 0.01 | Tukey HSD: Ranidae-Bufonidae p-adjusted = 0.008 |
| ***Bufonidae n = 19*** | 0.107 | 0.374 | 0.715 | 1.089 | 1.667 | 3.589 |  |  |
| ***Hylidae n = 25*** | 0.154 | 0.937 | 1.620 | 1.695 | 2.398 | 4.087 |  |  |
| ***Ranidae n = 148*** | 0.031 | 0.971 | 1.688 | 1.926 | 2.794 | 4.618 |  |  |
| ***Pr total n =10*** | 0.580 | 1.644 | 2.059 | 2.144 | 2.727 | 3.510 | Pr One-Way ANOVA: F(2, 7) = 1.22, p-value = 0.351 |  |
| ***Bufonidae n = 2*** | 1.414 | 1.559 | 1.705 | 1.705 | 1.850 | 1.996 |  |  |
| ***Hylidae n = 3*** | 0.580 | 1.262 | 1.945 | 1.754 | 2.342 | 2.739 |  |  |
| ***Ranidae n = 5*** | 1.543 | 2.122 | 2.691 | 2.554 | 2.904 | 3.51 |  |  |
| ***Rv total n = 29*** | 0.100 | 0.700 | 1.190 | 1.545 | 2.090 | 4.270 | Rv One-Way Welch ANOVA: F(2, 2.62) = 15.5, p-value = 0.035 | Two-tailed t-test: Hylidae-Ranidae p-value < 0.001 |
| ***Bufonidae n = 2*** | 1.140 | 1.202 | 1.265 | 1.265 | 1.327 | 1.390 |  |  |
| ***Hylidae n = 3*** | 0.650 | 0.660 | 0.670 | 0.673 | 0.685 | 0.700 |  |  |
| ***Ranidae n = 24*** | 0.100 | 0.893 | 1.475 | 1.678 | 2.393 | 4.270 |  |  |

**Supplemental Table S11. Distribution of pathogen intensity by family with associated parametric and non-parametric analysis of variance**.

| **Pathogen group** | **Min** | **1st_Qu** | **Median** | **Mean** | **3rd_Qu** | **Max** | **p-values**  **(α = 0.05)** | **Games-howell post-hoc (α = 0.05)** |
| --- | --- | --- | --- | --- | --- | --- | --- | --- |
| ***Bd total n = 192*** | 0.031 | 0.917 | 1.676 | 1.856 | 2.738 | 4.618 | Welch's ANOVA removing n < 5 samples: F(5,57.2) = 4.32, **p-value = 0.002** |  |
| ***A. americanus n = 15*** | 0.107 | 0.336 | 0.897 | 1.249 | 1.847 | 3.589 |  | *A. americanus* v. *R. clamitans* p-value = 0.026 |
| ***A. fowleri n = 2*** |  |  |  |  |  |  |  |  |
| ***A. terrestris n = 2*** |  |  |  |  |  |  |  |  |
| ***H. chrys/vers n = 5*** |  |  |  |  |  |  |  |  |
| ***H. cinerea n = 3*** |  |  |  |  |  |  |  |  |
| ***H. squirella n = 1*** |  |  |  |  |  |  |  |  |
| ***P. crucifer n = 16*** | 0.351 | 1.276 | 1.770 | 1.769 | 2.406 | 3.486 |  |  |
| ***R. catesbeiana n = 36*** | 0.207 | 0.926 | 1.529 | 1.468 | 1.809 | 4.154 |  | *R. catesbeiana* v. *R. clamitans* p value = 0.006 |
| ***R. clamitans n = 42*** | 0.031 | 1.224 | 2.470 | 2.339 | 3.304 | 4.618 |  |  |
| ***R. pipiens n = 16*** | 0.039 | 0.814 | 1.382 | 1.415 | 1.753 | 2.957 |  | *R. pipiens* v. *R. clamitans* p-value = 0.018 |
| ***R. sphenocephala n = 54*** | 0.076 | 0.962 | 2.004 | 2.035 | 2.945 | 4.180 |  |  |
| ***Pr total n = 10*** | 0.580 | 1.644 | 2.059 | 2.144 | 2.727 | 3.510 | Welch's ANOVA removing n < 2 samples: F(2,2.09) = 0.387, p-value = 0.72 |  |
| ***A. americanus n = 2*** | 1.414 | 1.559 | 1.705 | 1.705 | 1.850 | 1.996 |  |  |
| ***A. fowleri n = 0*** |  |  |  |  |  |  |  |  |
| ***A. terrestris n = 0*** |  |  |  |  |  |  |  |  |
| ***H. chrys/vers n = 2*** |  |  |  |  |  |  |  |  |
| ***H. cinerea n = 1*** |  |  |  |  |  |  |  |  |
| ***H. squirella n = 0*** |  |  |  |  |  |  |  |  |
| ***P. crucifer n = 0*** |  |  |  |  |  |  |  |  |
| ***R. catesbeiana n = 1*** |  |  |  |  |  |  |  |  |
| ***R. clamitans n = 1*** |  |  |  |  |  |  |  |  |
| ***R. pipiens n = 0*** |  |  |  |  |  |  |  |  |
| ***R. sphenocephala n = 3*** | 1.543 | 1.833 | 2.122 | 2.190 | 2.513 | 2.904 |  |  |
| ***Rv total n = 50*** | 0.100 | 0.700 | 1.190 | 1.545 | 2.090 | 4.270 |  |  |
| ***A. americanus n = 2*** | 1.140 | 1.202 | 1.265 | 1.265 | 1.327 | 1.390 |  |  |
| ***A. fowleri n = 0*** |  |  |  |  |  |  |  |  |
| ***A. terrestris n = 0*** |  |  |  |  |  |  |  |  |
| ***H. chrys/vers n = 1*** |  |  |  |  |  |  |  |  |
| ***H. cinerea n = 2*** | 0.650 | 0.663 | 0.675 | 0.675 | 0.688 | 0.700 |  |  |
| ***H. squirella n = 0*** |  |  |  |  |  |  |  |  |
| ***P. crucifer n = 0*** |  |  |  |  |  |  |  |  |
| ***R. catesbeiana n = 15*** | 0.200 | 0.955 | 1.770 | 1.802 | 2.525 | 4.270 | t(20) = 0.566, p-value = 0.584 |  |
| ***R. clamitans n = 7*** | 0.100 | 0.685 | 1.350 | 1.467 | 1.680 | 4.090 |  |  |
| ***R. pipiens n = 0*** |  |  |  |  |  |  |  |  |
| ***R. sphenocephala n = 3*** | 1.543 | 1.833 | 2.122 | 2.190 | 2.513 | 2.904 |  |  |

| **Pathogen** | **N individual** | **Age class** | **+** | **Min.** | **1st Qu.** | **Median** | **Mean** | **3rd Qu.** | **Max** | **Two-tailed t-test (α = 0.05)** |
| --- | --- | --- | --- | --- | --- | --- | --- | --- | --- | --- |
| Bd | 192 | adult | 0.03 | 0.85 | 1.53 | 1.65 | 2.37 | 4.18 | 0.03 | t(156) = -3.63, p-value = 0.004 |
|  |  | juvenile | 0.30 | 1.32 | 2.51 | 2.62 | 3.61 | 4.62 | 0.30 |  |
|  |  | unknown | 0.08 | 0.74 | 1.78 | 1.87 | 2.90 | 4.15 | 0.08 |  |
| Pr | 10 | adult | 0.58 | 1.41 | 2.00 | 1.88 | 2.69 | 2.74 | 0.58 |  |
|  |  | juvenile | 3.51 | 3.51 | 3.51 | 3.51 | 3.51 | 3.51 | 3.51 |  |
|  |  | unknown | 1.54 | 1.84 | 2.03 | 2.13 | 2.32 | 2.90 | 1.54 |  |
| Rv | 29 | adult | 0.10 | 0.61 | 1.20 | 1.50 | 2.09 | 4.27 | 0.10 | t(21)=-0.25, p-value = 0.807 |
|  |  | juvenile | 0.20 | 1.08 | 1.19 | 1.64 | 1.94 | 4.09 | 0.20 |  |
|  |  | unknown | 0.70 | 0.92 | 1.42 | 1.55 | 2.01 | 2.81 | 0.70 |  |

**Supplemental Table S12. Distribution of pathogen intensity by species with associated parametric and non-parametric analyses of difference of means and variance**.

**Supplemental Table S13. Summary of the distribution of pathogen intensity for infected individuals across age class.** Intensity values are represented by the log transformed SQ value. Two-tailed t-test results show the comparison of mean Bd and Rv intensities between adult and juvenile frogs at an alpha value of 0.05.

| **Pathogen** | **N individual** | **Sex** | **+** | **Min.** | **1st Qu.** | **Median** | **Mean** | **3rd Qu.** | **Max** | **Two-tailed t-test (α = 0.05** |
| --- | --- | --- | --- | --- | --- | --- | --- | --- | --- | --- |
| Bd | 192 | female | 43 | 0.03 | 0.86 | 1.53 | 1.58 | 2.16 | 4.18 | t(105) = -0.723, p-value = 0.472 |
|  |  | male | 64 | 0.15 | 0.84 | 1.58 | 1.73 | 2.48 | 4.14 |  |
|  |  | unknown | 85 | 0.04 | 0.93 | 1.84 | 2.00 | 2.90 | 4.62 |  |
| Pr | 10 | female | 2 | 1.41 | 1.56 | 1.71 | 1.71 | 1.85 | 2.00 |  |
|  |  | male | 3 | 0.58 | 1.64 | 2.69 | 2.00 | 2.72 | 2.74 |  |
|  |  | unknown | 5 | 1.54 | 1.94 | 2.12 | 2.41 | 2.90 | 3.51 |  |
| Rv | 29 | female | 2 | 0.65 | 1.41 | 2.17 | 2.17 | 2.92 | 3.68 | t(10) = 1.3, p-value = 0.222 |
|  |  | male | 10 | 0.10 | 0.45 | 0.79 | 1.07 | 1.75 | 2.53 |  |
|  |  | unknown | 17 | 0.20 | 1.07 | 1.39 | 1.76 | 2.09 | 4.27 |  |
|  |  |  |  |  |  |  |  |  |  |  |

**Supplemental Table S14. Summary of the distribution of pathogen intensity for infected individuals across sexes.** Intensity values are represented by the log transformed SQ value. Two-tailed t-test results show the comparison of mean Bd and Rv intensities between female and male frogs at an alpha value of 0.05.
