# Supplementary figures and images for: Leveraging machine learning to uncover multi-pathogen infection dynamics across co-distributed frog families
Source: PeerJ. 2025 Jan 29;13:e18901. doi: 10.7717/peerj.18901 (PMC11786709; doi:10.7717/peerj.18901)

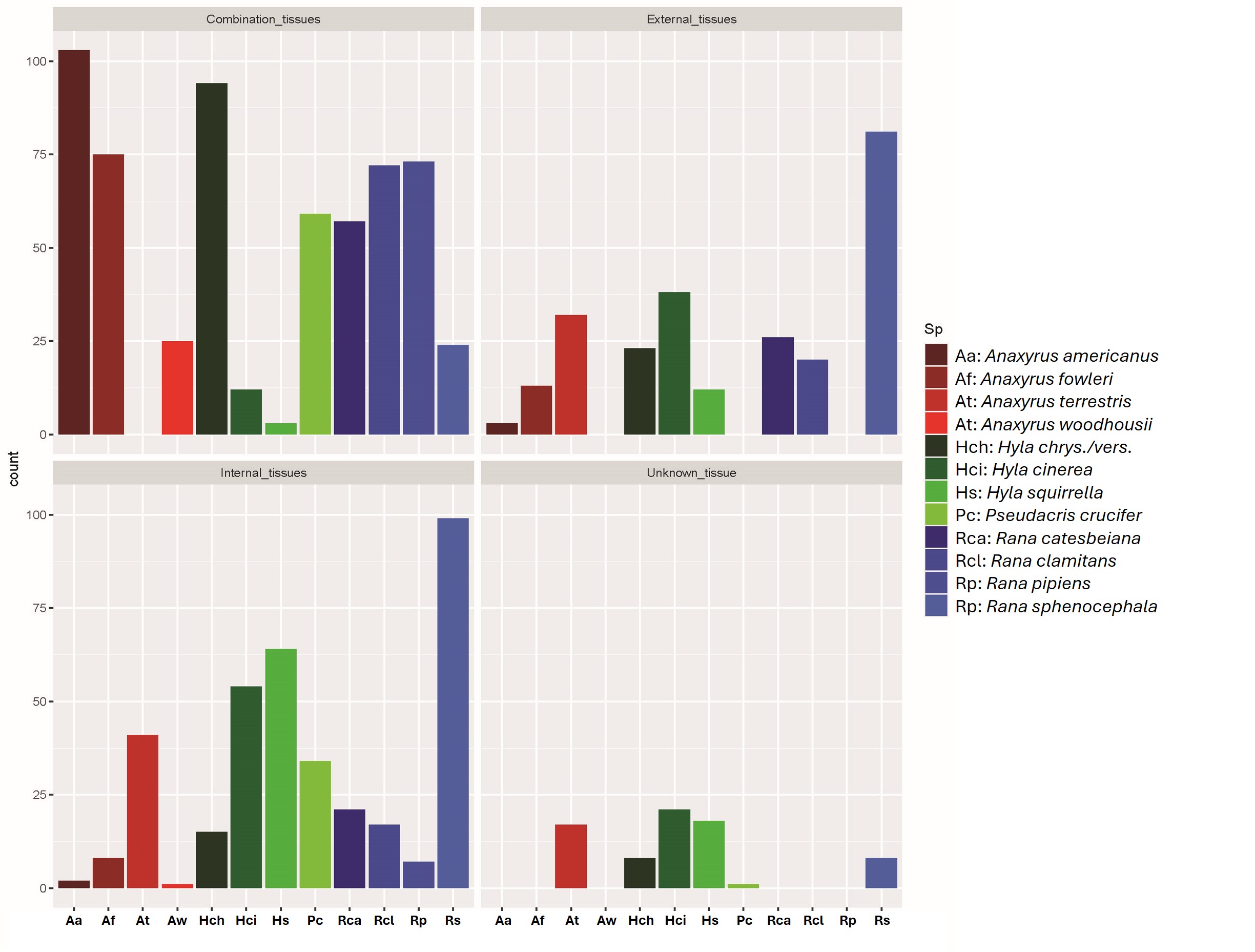

Supplement: Supplemental Information 3 — Individual counts for tissue type extracted and used for Bd screening are shown for each species. Stars denote species found in the southeastern U.S. region that were sampled prior to 2021. Combination tissues include both internal (muscle, liver) and external (toe/tail, including skin) tissue. [file peerj-13-18901-s003.jpg]

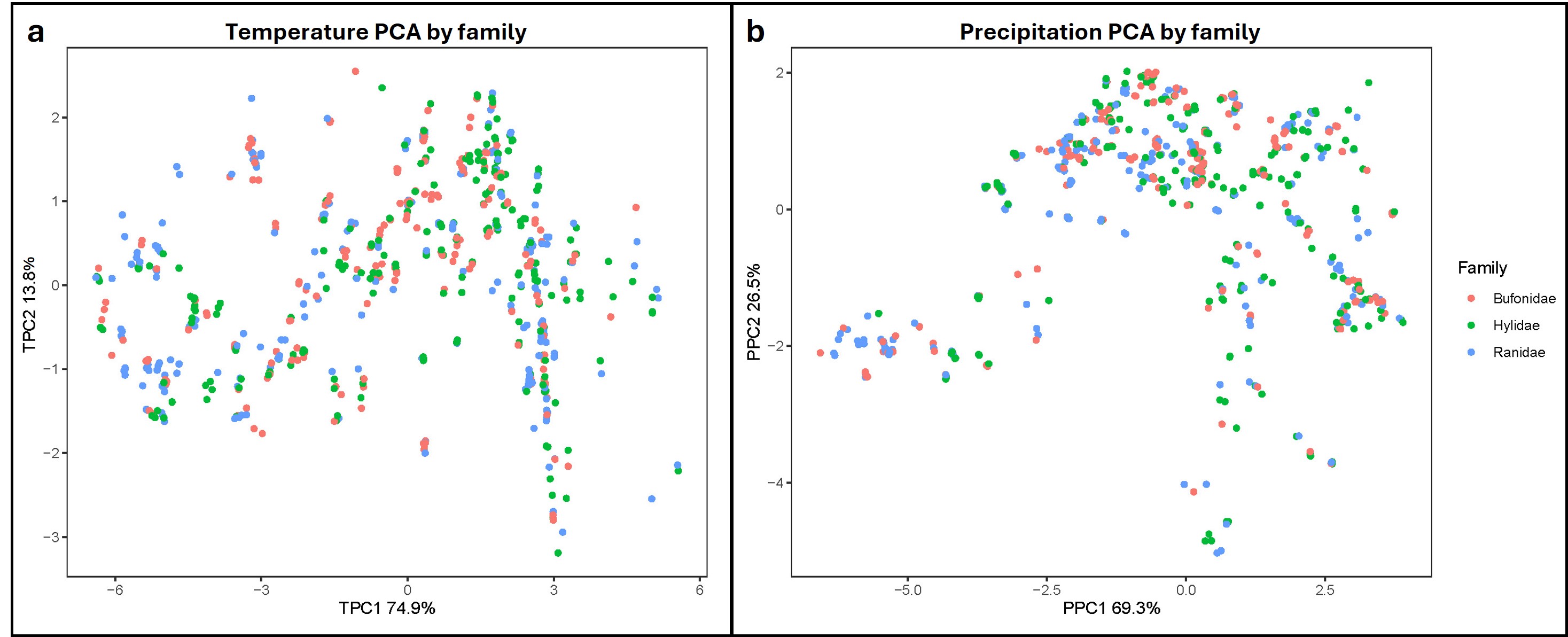

Supplement: Supplemental Information 4 — Individual points are colored by family and represent values along the first and second PC dimensions which represent 11 bioclimatic variables relating to temperature (left) and 9 bioclimatic variables relating to precipitation (right). [file peerj-13-18901-s004.jpg]

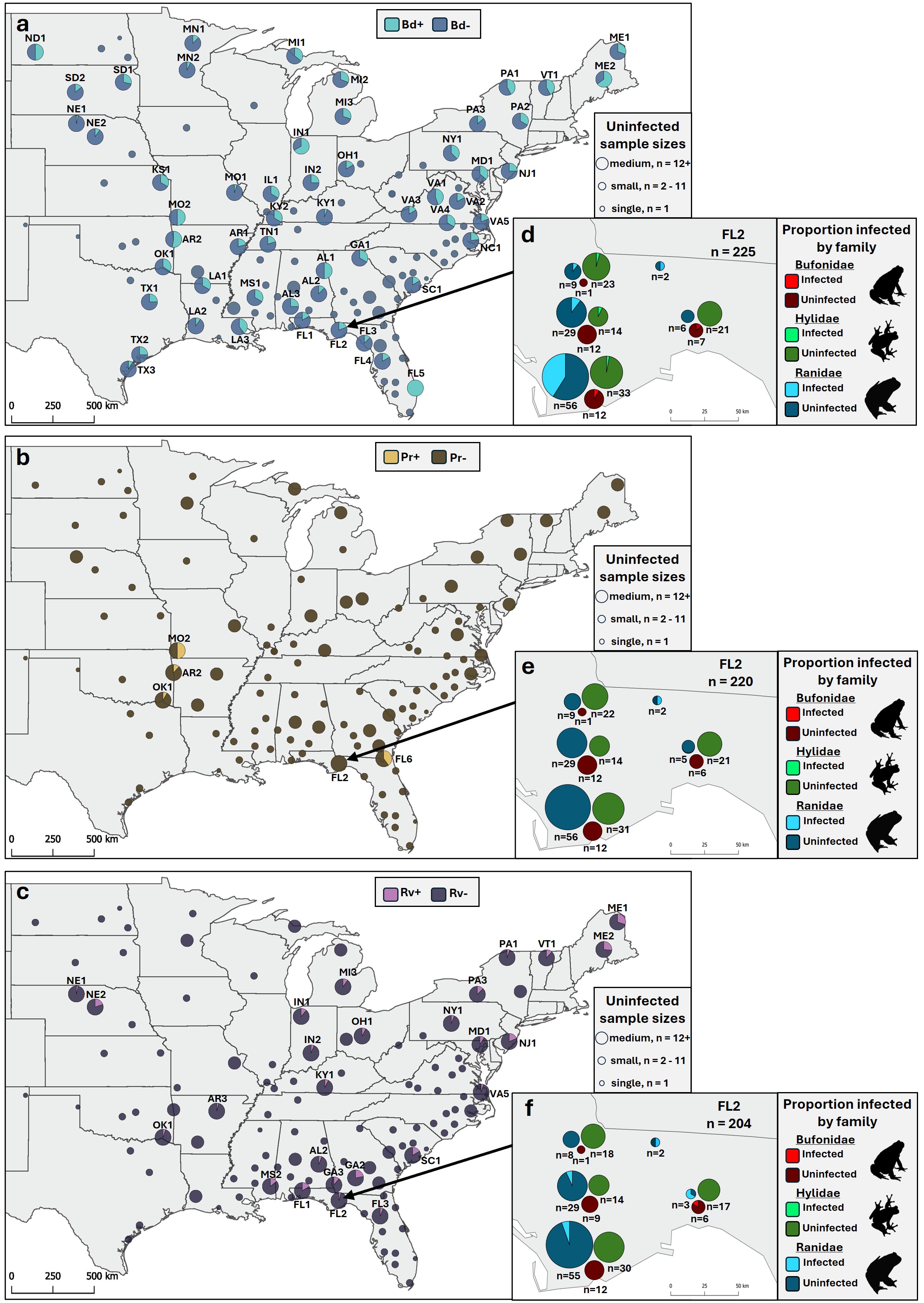

Supplement: Supplemental Information 5 — Pie charts represent the proportion of infected individuals pooled within a 50 km radius for (a, d) Bd, (b, e) Pr, and (c, f) Rv. Uninfected sites are pooled within a 100 km radius and circle sizes reflect the number of individuals at that site. (d–f) Proportion of infected individuals at the community level, pooled at <50 km for our densest sampling site (FL2) with number of individuals below. [file peerj-13-18901-s005.jpg]

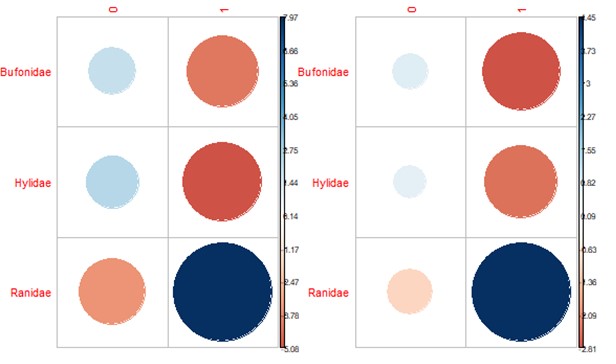

Supplement: Supplemental Information 6 — Correlation plots show the direction and contribution to Pearson’s residuals of Bd (left) and Rv (right) infection counts by family. Positive residuals are blue, suggesting a positive association between the corresponding row and column, and negative residuals are red, suggesting a negative association. Size of the circle indicates relative contribution to Pearson’s residual. [file peerj-13-18901-s006.jpg]

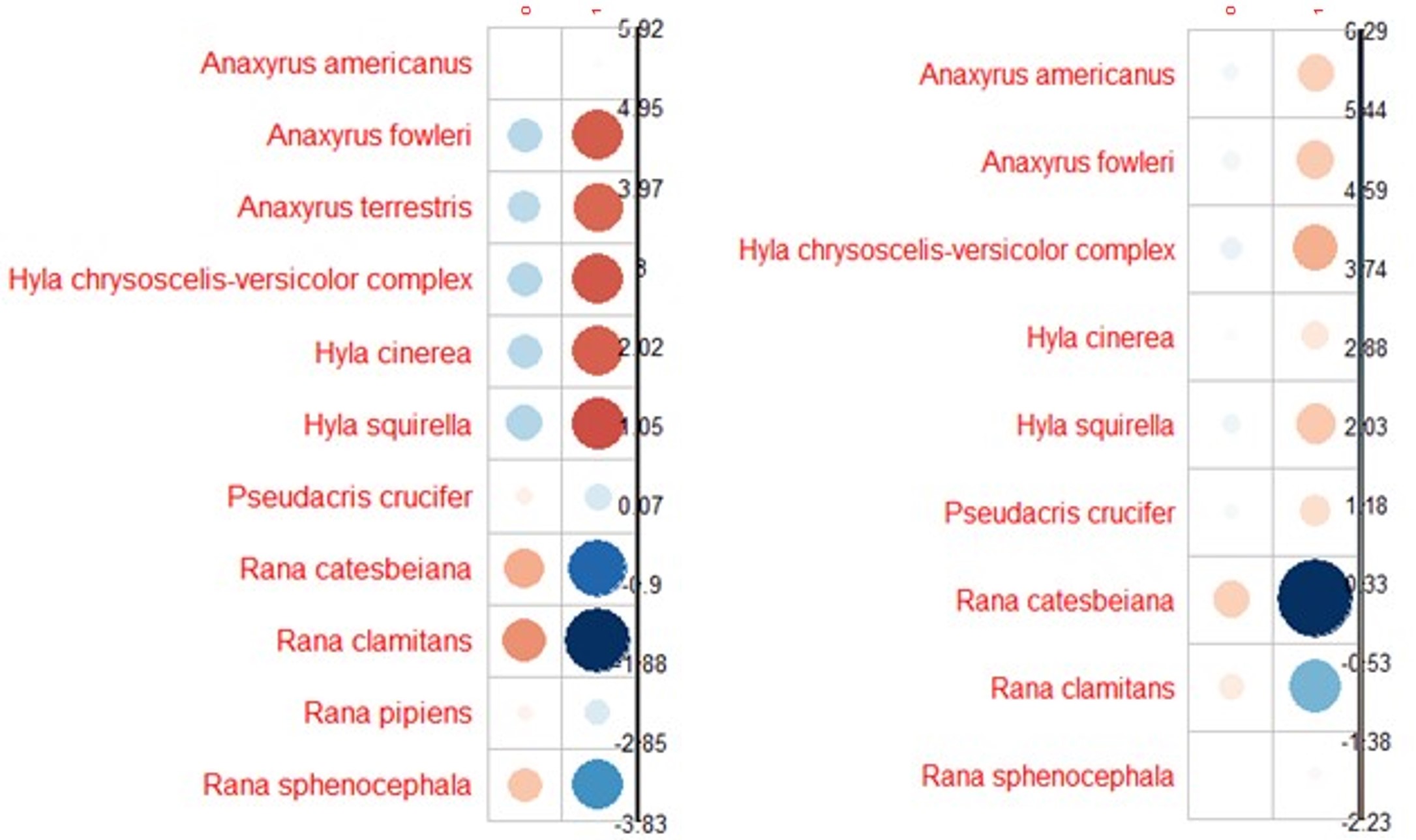

Supplement: Supplemental Information 7 — Correlation plots show the direction and contribution to Pearson’s residuals of Bd (left) and Rv (right) infection counts by species. Positive residuals are blue, suggesting a positive association between the corresponding row and column, and negative residuals are red, suggesting a negative association. Size of the circle indicates relative contribution to Pearson’s residual. [file peerj-13-18901-s007.jpg]

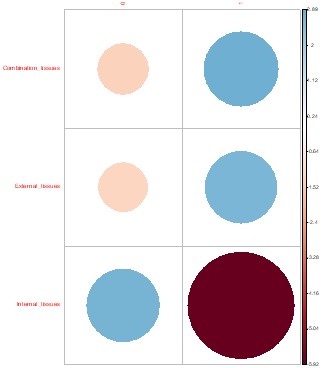

Supplement: Supplemental Information 8 — Correlation plots show the direction and contribution to Pearson’s residuals of Bd infection counts by tissue types used in DNA extraction. Positive residuals are blue, suggesting a positive association between the corresponding row and column, and negative residuals are red, suggesting a negative association. Size of the circle indicates relative contribution to Pearson’s residual. [file peerj-13-18901-s008.jpg]

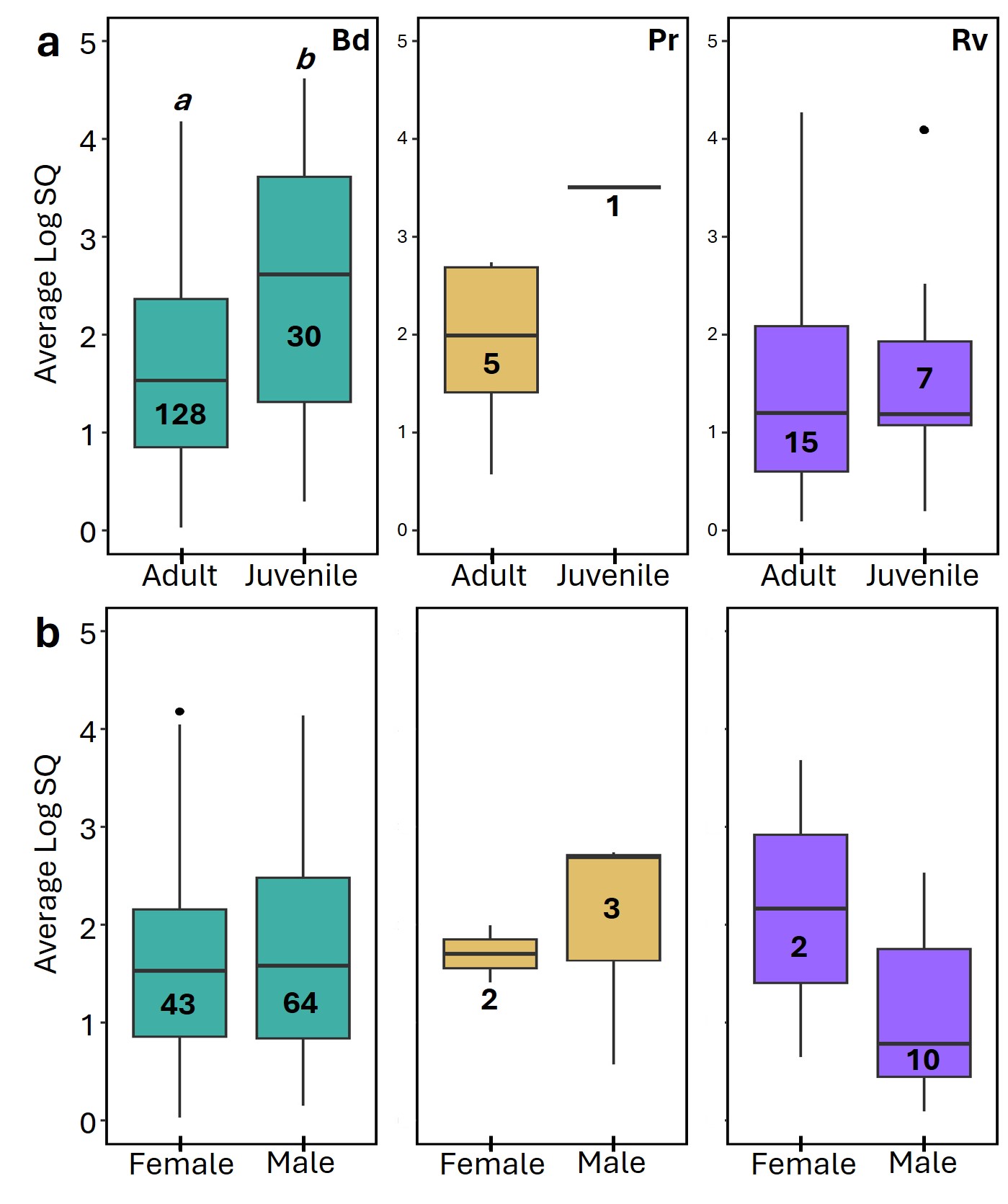

Supplement: Supplemental Information 9 — Infection intensity is measured in log(SQ) across (a) age classes and (b) sex for Bd (left), Pr (center), and Rv (right) infections. Significant differences (p < 0.05) between means for each paired comparison are indicated by differing letters (a, b). [file peerj-13-18901-s009.jpg]

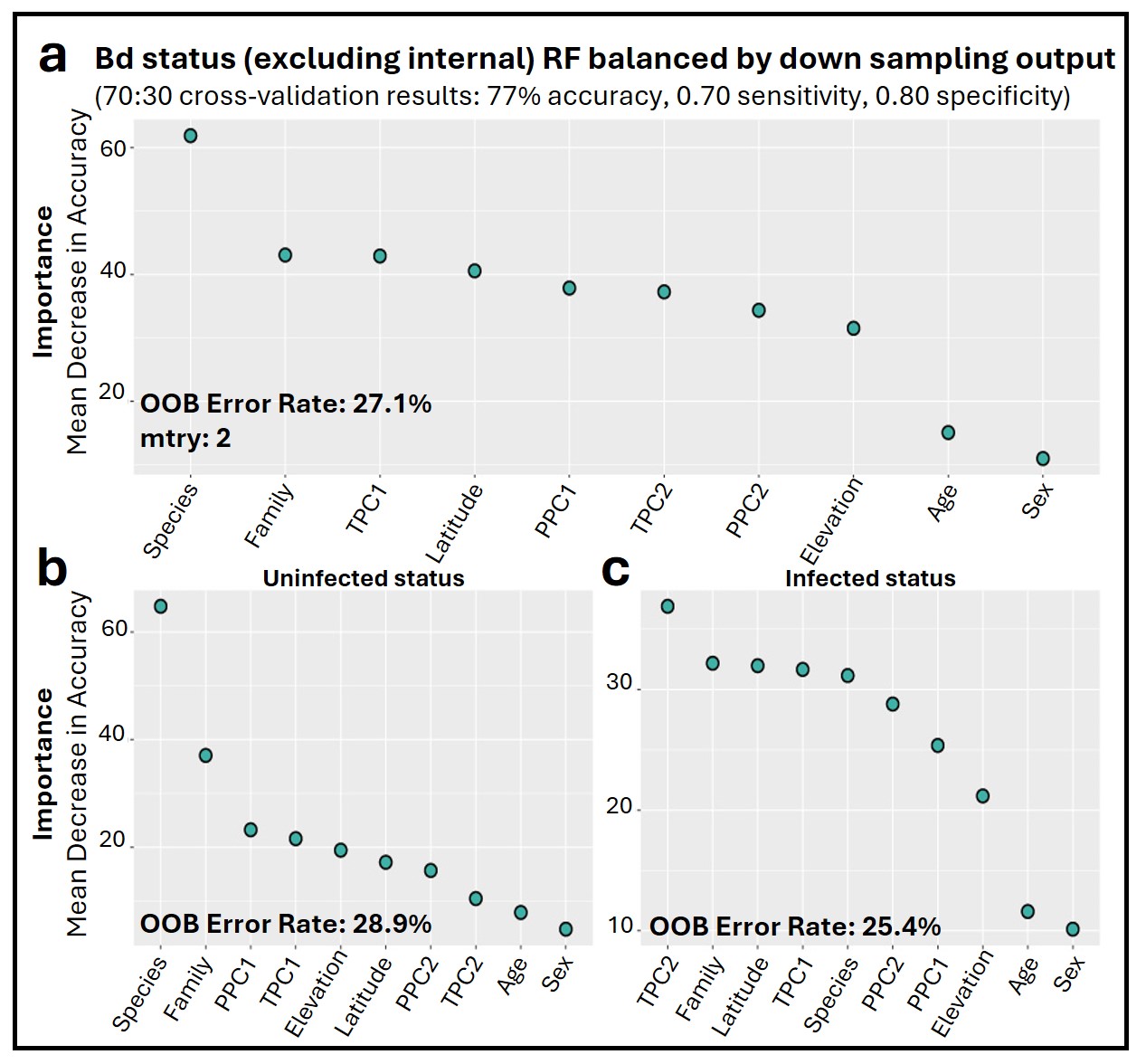

Supplement: Supplemental Information 10 — (a) Overall model of Bd infection status. (b-c) Variables ranked by importance for Bd uninfected and infected status, respectively. For all panels, variable importance is measured as mean decrease in accuracy, averaged across 100 iterations, and ranked from highest (left) to lowest (right) as determined by balanced classification RF analyses. Average out-of-bag (OOB) classification error rates are shown. Predictive accuracy, sensitivity, and specificity values of the final models were derived from cross-validation, with 70% of the data used for training and 30% for testing. [file peerj-13-18901-s010.jpg]

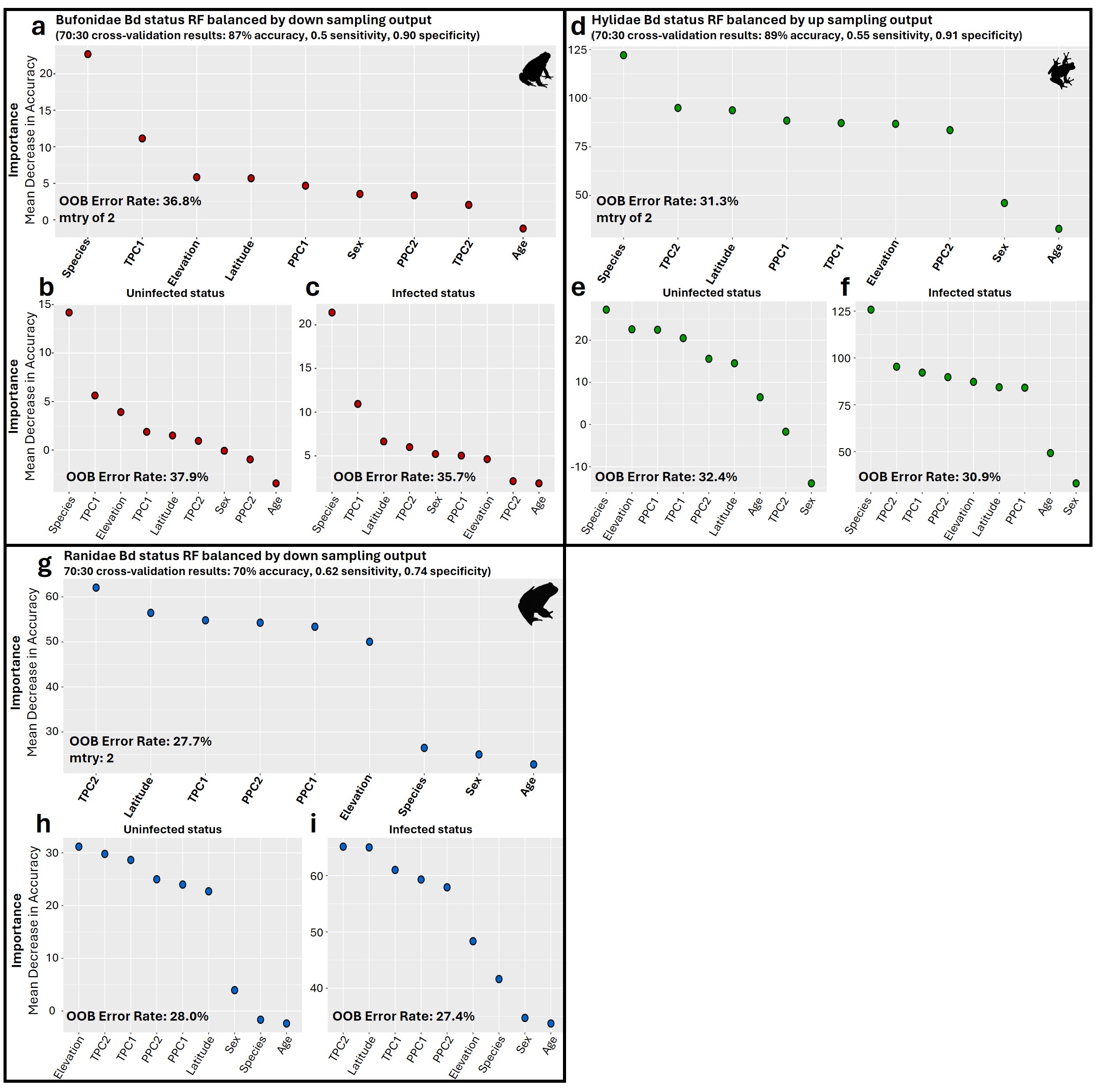

Supplement: Supplemental Information 11 — (a) Overall model of Bd infection status for Bd_Bufonidae models only. (b, c) Variables ranked by importance for Bd uninfected and infected status, respectively. (d) Overall model of Bd infection status for Bd_Hylidae models only. (e, f) Variables important for Bd uninfected and infected status, respectively. (g) Overall model of Bd infection status for Bd_Ranidae models only. (h, i) Variables important for Bd uninfected and infected status, respectively. For all panels, variable importance is measured as mean decrease in accuracy, averaged across 100 iterations, and ranked from highest (left) to lowest (right) as determined by balanced classification RF analyses. Average out-of-bag (OOB) classification error rates are shown. Predictive accuracy, sensitivity, and specificity values of the final models were derived from cross-validation, with 70% of the data used for training and 30% for testing. [file peerj-13-18901-s011.jpg]

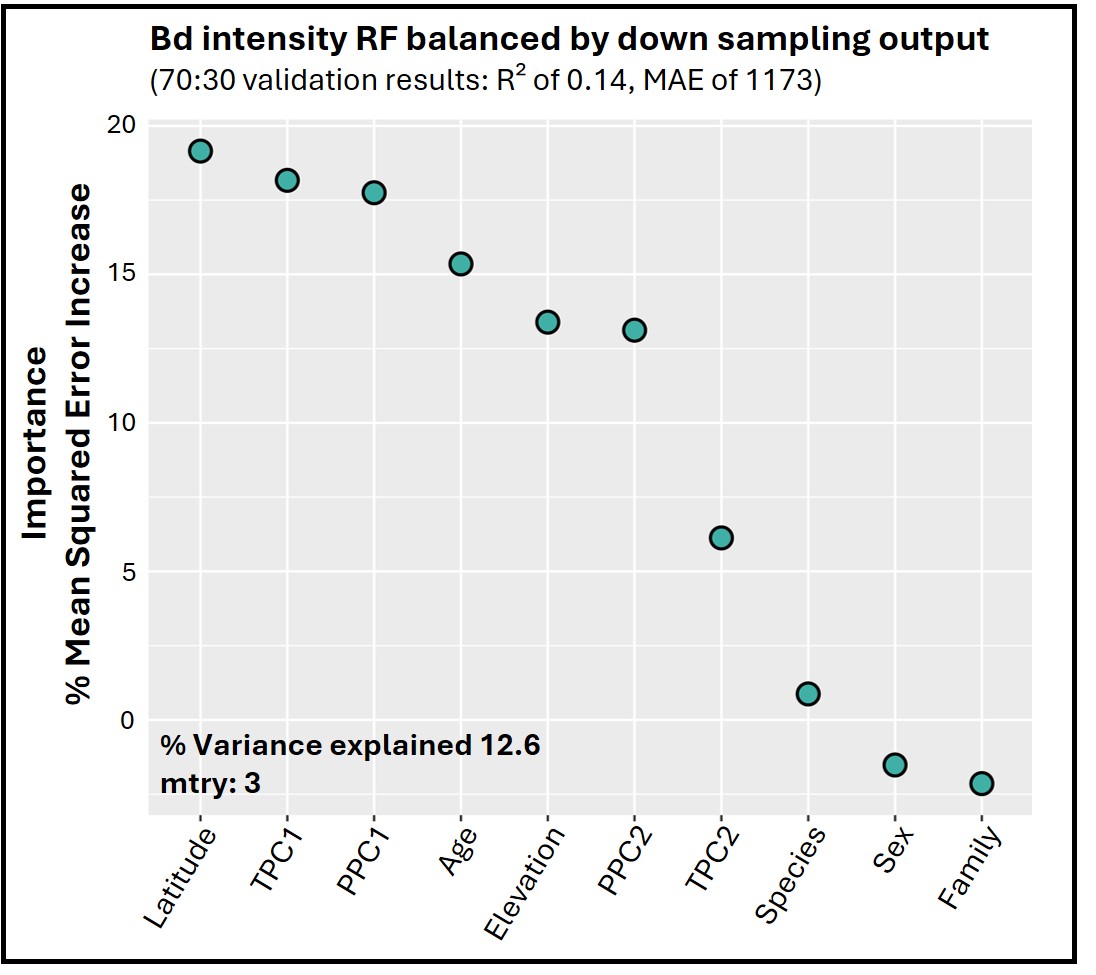

Supplement: Supplemental Information 12 — Variable importance is measured as percentage mean squared error increase decrease, averaged across 100 iterations, and ranked from highest (left) to lowest (right) as determined by RF regression analysis. Percent variance explained is shown. R2 and mean absolute error (MAE) were derived from model validation, with 70% of the data used for training and 30% for testing. [file peerj-13-18901-s012.jpg]

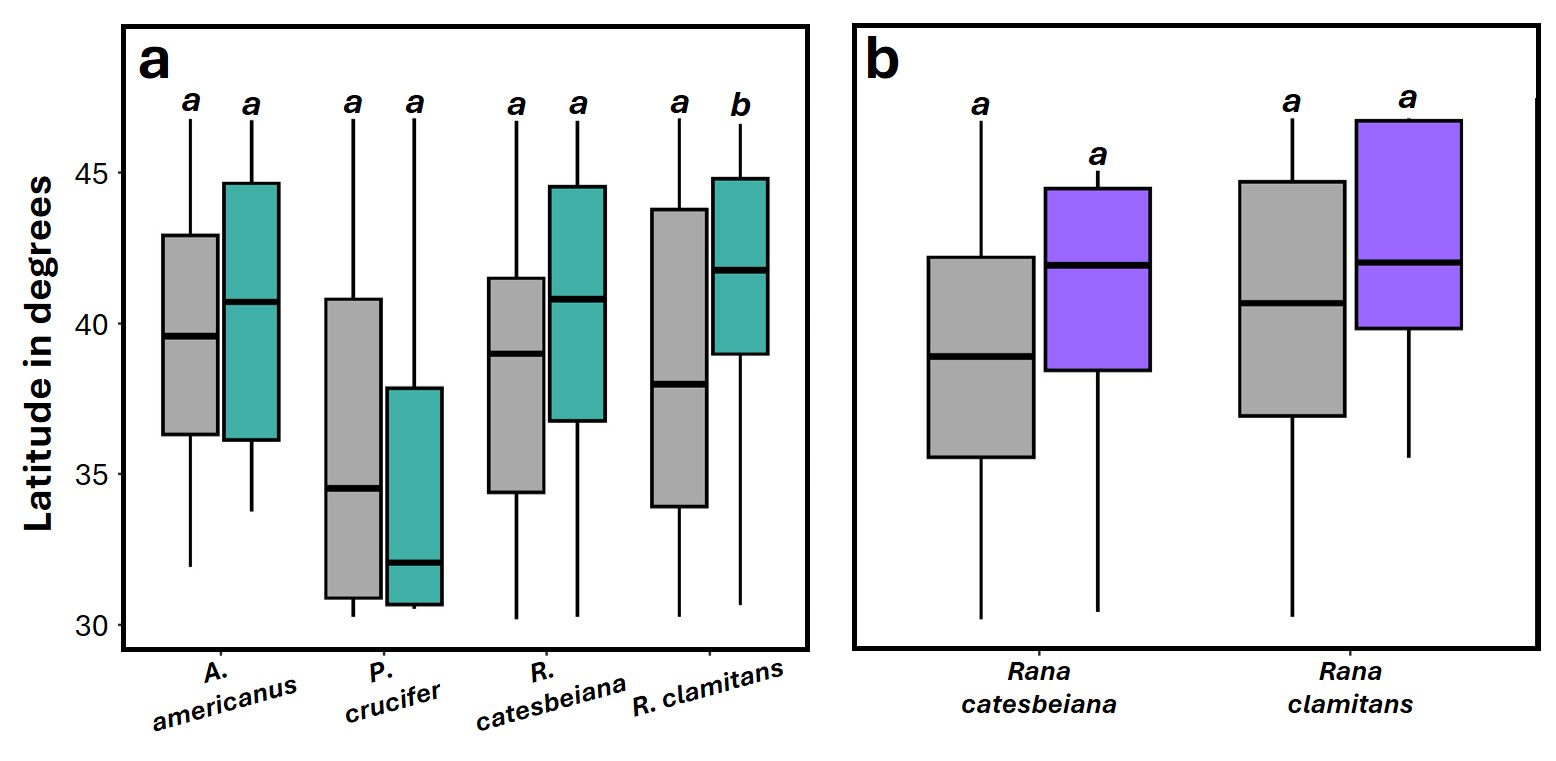

Supplement: Supplemental Information 13 — (a) Species with adequate number of Bd infections (n > 15 positive individuals per species) and (b) species with adequate number of Rv infections (n > 2 positive individuals per species). Significant differences (p < 0.05) between means for each paired comparison are indicated by differing letters (a, b). [file peerj-13-18901-s013.jpg]
